# Differential MicroRNA Signatures in the Pathogenesis of Barrett's Esophagus

**DOI:** 10.14309/ctg.0000000000000125

**Published:** 2020-01-13

**Authors:** Michael P. Craig, Sumudu Rajakaruna, Oleg Paliy, Mumtaz Sajjad, Srivats Madhavan, Nikhil Reddy, Jin Zhang, Michael Bottomley, Sangeeta Agrawal, Madhavi P. Kadakia

**Affiliations:** 1 Department of Biochemistry and Molecular Biology, Boonshoft School of Medicine, Wright State University, Dayton, Ohio, USA;; 2 Dayton VA Medical Center, Dayton, Ohio, USA;; 3 Statistical Consulting Center, Wright State University, Dayton, Ohio, USA;; 4 Department of Internal Medicine, Boonshoft School of Medicine, Wright State University, Dayton, Ohio, USA.

## Abstract

**OBJECTIVES:**

Barrett's esophagus (BE) is the precursor lesion and a major risk factor for esophageal adenocarcinoma (EAC). Although patients with BE undergo routine endoscopic surveillance, current screening methodologies have proven ineffective at identifying individuals at risk of EAC. Since microRNAs (miRNAs) have potential diagnostic and prognostic value as disease biomarkers, we sought to identify an miRNA signature of BE and EAC.

**METHODS:**

High-throughput sequencing of miRNAs was performed on serum and tissue biopsies from 31 patients identified either as normal, gastroesophageal reflux disease (GERD), BE, BE with low-grade dysplasia (LGD), or EAC. Logistic regression modeling of miRNA profiles with Lasso regularization was used to identify discriminating miRNA. Quantitative reverse transcription polymerase chain reaction was used to validate changes in miRNA expression using 46 formalin-fixed, paraffin-embedded specimens obtained from normal, GERD, BE, BE with LGD or HGD, and EAC subjects.

**RESULTS:**

A 3-class predictive model was able to classify tissue samples into normal, GERD/BE, or LGD/EAC classes with an accuracy of 80%. Sixteen miRNAs were identified that predicted 1 of the 3 classes. Our analysis confirmed previous reports indicating that miR-29c-3p and miR-193b-5p expressions are altered in BE and EAC and identified miR-4485-5p as a novel biomarker of esophageal dysplasia. Quantitative reverse transcription polymerase chain reaction validated 11 of 16 discriminating miRNAs.

**DISCUSSION:**

Our data provide an miRNA signature of normal, precancerous, and cancerous tissue that may stratify patients at risk of progressing to EAC. We found that serum miRNAs have a limited ability to distinguish between disease states, thus limiting their potential utility in early disease detection.

## INTRODUCTION

Barrett's esophagus (BE) is a metaplastic lesion that develops in the distal esophagus in response to chronic gastroesophageal reflux (GERD). BE is characterized by replacement of the normal squamous epithelium of the distal esophagus by an intestinal-like columnar epithelium. BE is known to be the premalignant precursor for adenocarcinomas of the esophagus and is associated with a 30- to 125-fold increase in risk of developing esophageal adenocarcinoma (EAC) (1,2). Once diagnosed, EAC has a 5-year survival rate of 17%–22% (3,4), thus demonstrating the critical need for early detection of patients at risk of developing EAC. Patients with BE are monitored for dysplasia using endoscopy, cell-sampling cytology balloons, or cytosponge (5–7), but since only 0.1%–4% progress to EAC (8–11), the cost-effectiveness of surveillance is controversial. Efforts are underway to identify biomarkers with diagnostic or prognostic potential in esophageal cancer.

MicroRNAs (miRNAs) are small noncoding RNAs that regulate gene expression. Cancers have distinct miRNA profiles (12), and miRNA biomarkers have been identified for the early detection of gastric, hepatocellular, breast, and non–small cell lung cancers (13–16). In addition, miRNAs are shown to stratify prostate cancer risk (17,18), predict recurrence, and survival in melanoma (19) and aid in the diagnosis, therapy, and prognosis of gastric cancer (20). At least 105 miRNAs are differentially regulated in BE vs normal controls (21). MiR-133a-3p, 136-5p, 194-5p, 382-5p, and 451a are dysregulated in serum from patients with BE and can differentiate between controls, BE, and EAC patients (22). In addition, miR-192, 194, 203, 205, and 215 have been identified as promising tissue biomarkers for the diagnosis and monitoring of BE (23).

In this study, high-throughput sequencing (HTS) of serum and tissue biopsy specimens obtained from normal subjects, patients with GERD, BE, BE with low-grade dysplasia (LGD), or EAC was performed to identify miRNA biomarkers specific to disease stage. Logistic regression modeling was used to identify 16 miRNAs which can categorize samples into either normal, BE/GERD, or LGD/EAC. The relative expression of 11/16 miRNAs was confirmed by quantitative reverse transcription polymerase chain reaction (qRT-PCR) using formalin-fixed, paraffin-embedded (FFPE) samples. These data provide an miRNA signature of normal, precancerous, and cancerous tissue that may stratify patients at risk of progressing to EAC. Furthermore, we identified miR-4485-5p as a novel biomarker of esophageal dysplasia.

## MATERIALS AND METHODS

### Ethics statement

This study was approved by the institutional regulatory board (IRB) of the Dayton Veterans Affairs Medical Center. Informed consent was obtained from all patients before obtaining serum and tissue samples.

### Study subjects

Study participants were randomly chosen from individuals who underwent esophageal biopsies at the Dayton VA hospital. Serum was obtained from 5 normal, 9 GERD, 7 BE, 5 BE with LGD, and 5 EAC subjects enrolled in the study and was kept at −80 °C. Tissue biopsies were obtained during high-definition/high-resolution white light endoscopy per the American College of Gastroenterologists guidelines using the Seattle biopsy protocol. Specifically, four-quadrant biopsies at 2-cm intervals were collected in patients without dysplasia and 1-cm interval in patients with previous dysplasia. Tissue biopsies stored in RNA later were obtained from 4 normal, 7 GERD, 7 BE, 5 LGD, and 5 EAC subjects enrolled in the study. H&E sections from each sample were interpreted by an expert gastrointestinal histopathologist. A summary of the demographic data for these samples is provided in Table 1 and detailed in Supplemental Table 1, Supplementary Digital Content 1, http://links.lww.com/CTG/A160.

**Table 1.**
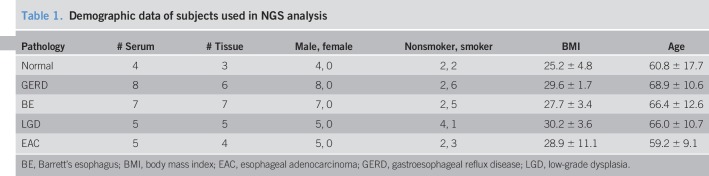
Demographic data of subjects used in NGS analysis

Study samples used for qRT-PCR validation of miRNA levels were randomly selected from archived deidentified FFPE tissues. A total of 15 normal, 16 GERD/BE, and 15 LGD/HGD/EAC samples were collected. The associated subject demographic data for these samples are summarized in Table 2 and detailed in Supplemental Table 2, Supplementary Digital Content 2, http://links.lww.com/CTG/A161. Although the amount of “time with BE” is a known risk factor for EAC progression, it is important to point out that the utility of this metric of progression to EAC varies greatly between individuals, thus making it impossible to place an individual on the continuum as “early BE” or “advanced BE.”

**Table 2.**
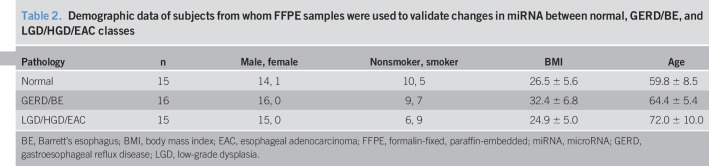
Demographic data of subjects from whom FFPE samples were used to validate changes in miRNA between normal, GERD/BE, and LGD/HGD/EAC classes

### High-throughput sequencing

Small RNAs isolated from serum and homogenized tissue using the mirVanaTM ParisTM RNA and Native Protein Purification kit (Thermo Fisher Scientific, Waltham, MA) were subjected to library preparation using the Ion Total RNA-seq v2 kit (Thermo Fisher Scientific) (24). Samples were sequenced using the Ion Proton system, Ion P1 chip, and Ion PI HI-Q Sequencing 200 kit (Life Technologies, Carlsbad, CA) followed by data analysis using Partek Flow software v7.0 (Partek, St. Louis, MO) with the miRBase mature miRNAs version 21 reference as previously described (24).

### Statistical analyses of miRNA data set

Because sequencing depth influences the probability of detection of low-abundance transcripts, samples were rarefied to 276,919 reads each. Since read density analysis indicated that read distribution in all samples was not normal, all values were square-root–transformed to compress the expression range and improve data normality (25). Loess transformation was then used to normalize the distribution of miRNA expression values among all samples as previously reported (26). This rarefied, square-root–transformed, Loess-normalized data set was then subjected to multivariate ordination analyses (27). For discriminant analyses, miRNA variables with stable expression values across all sample types were filtered out which led to the reduction of the data set from 2,555 to 683 miRNA species. Logistic regression with Lasso regularization (LR:LR) (28) was chosen to generate a sample classification model. Lasso regularization allowed us to limit the number of discriminatory variables defining each sample type. The logistic regression with Lasso regularization was the primary model used because it allowed us to limit the number of miRNA markers defining each class. Because of the ability of LR-LR classifier to use a limited number of miRNA variables in its class prediction, this approach was chosen initially and not because it had the best performance. The LR:LR method achieved the highest accuracy of class prediction compared with Random Forest, Support Vector Machines, and K-Nearest Neighbor classifiers (data not shown). These other models were run for comparison purposes to ensure that Lasso regularization did not compromise model accuracy and performance.

### qRT-PCR from FFPE sections

Total RNA was isolated from 25-µm FFPE tissue sections using the truXTRAC FFPE microTube RNA kit and M220 focused ultrasonicator (Covaris, Woburn, MA) following manufacturer protocols. Approximately 10 ng of total RNA was converted to cDNA using the TaqMan Advanced miRNA cDNA KIT and amplified in a 14-cycle miR-AMP reaction per manufacturer recommendations (Life Technologies). qRT-PCR was performed on a QuantStudio 7 (Applied Biosystems, Foster City, CA) with each sample run in triplicate. qRT-PCR was performed using TaqMan Fast Advanced Master Mix (Life Technologies) and miRNA-specific AODS as follows: hsa-miR-93-5p (478210_mir), hsa-miR-423-5p (478090_miR), hsa-let-7g-5p (478580_miR), hsa-miR-29c-3p (479229_mir), hsa-miR-30d-5p (478606_mir), hsa-miR-34b-3p (478050_mir), hsa-miR-106b-3p (478412_mir), hsa-miR-133a-3p (478511_mir), hsa-miR-193-3p (478314_mir), hsa-miR-203a-5p (478756_mir), hsa-miR-203b-5 (478758_mir), hsa-miR-212-5p (478767_miR), hsa-miR-369-5p (478068_mir), hsa-miR-375-3p (478074_mir), hsa-miR-381-3p (477816_mir), hsa-miR-4783-5p (47944_miR), hsa-miR-4485-5p (480829_miR), and hsa-miR-4792 (480052_miR). Analysis was performed using Relative Quantification v3.8 (Thermo Fisher Cloud utility). Raw CT values were normalized to endogenous control miRNAs hsa-miR-93-5p and hsa-miR-423-5p, both recommended in the TaqMan Advanced miRNA protocol (Thermo Fisher Scientific, Carlsbad, CA) and displaying comparable expression across all samples (data not shown). Relative expression presented as 2^−ΔCT^.

### Statistical analysis of Reverse Transcription Quantitative Polymerase Chain Reaction data

Independent-samples, 2-tailed *t* tests for equal or unequal variance were performed to test for significant differences in relative expression (2^−ΔCT^) in the predicted class vs all other samples. Differences were considered statistically significant at *P* < 0.05. Relative expression values that were not normally distributed were assessed using the Wilcoxon two-sample test. SAS version 9.4 (SAS Institute, Cary, NC) was used for all analyses.

### Ingenuity pathway analysis (IPA)

Experimentally validated human mRNA targets for 16 predictive miRNAs were identified using the Ingenuity Pathways Knowledge Base (Qiagen, Valencia, CA). The algorithms used in IPA (Qiagen) have been previously described (29). Identified mRNA targets were compared with the IPA Knowledge Base lists of mRNA associated with BE and EAC. Functional analysis of target mRNA was performed using IPA pathway tools.

## RESULTS

### miRNA expression profiles determined by serum or tissue source

**Figure 1.**
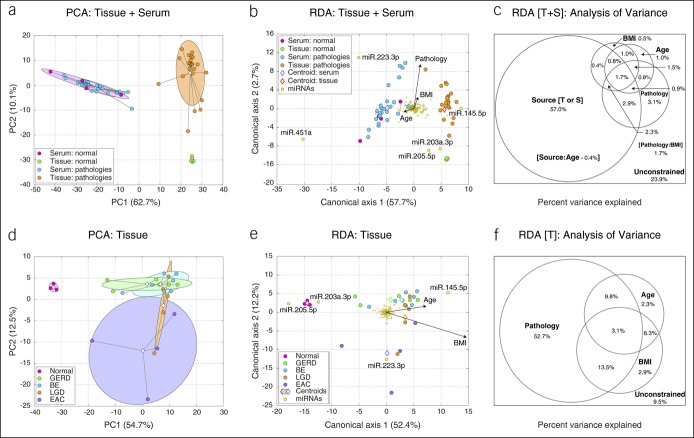
Comparison of miRNA expression profiles from tissue and serum samples. Similarity among all samples was assessed by unconstrained principal component analysis (PCA, **a**, **d**) and constrained redundancy analysis (RDA, **b**, **e**) run on the square-root–transformed, Loess-normalized miRNA expression data set. Both tissue and serum samples were included in analyses visualized in (**a**, **b**). (**d** and **e)** Visualize outputs of PCA and RDA ran on tissue samples exclusively. Each sample is shown as circle colored according to the sample type (see legend). The percent of data set variability explained by each axis is shown in parentheses in axis titles. In (**a**, **d**), group clouds represent areas of 3 standard errors around the group centroid (diamond). Arrows in RDA triplot denote the magnitude and the direction of the effect of constraining continuous variables. For constraining categorical variables, each class centroid is shown as a diamond. miRNAs that were associated strongly with particular sample groups are named. Analysis of variance diagrams (**c**, **f**) depict the relative contribution of constraining variables to the overall variability in the data set. miRNA, microRNA.

Principal component analysis of the HTS data set obtained from serum and tissue samples from 31 subjects indicated a clear separation of serum and tissue samples (Figure 1a). Although normal and pathological tissue samples formed distinct clusters, no separation was observed between normal and pathological serum samples. These findings were confirmed by redundancy analysis (RDA) in which miRNA variability was constrained by several explanatory variables including sample source (serum or tissue), sample type (normal or pathology), body mass index (BMI), and age (Figure 1b). Analysis of variance indicated that sample source was the dominant determinant of the miRNA expression, while other variables contributed substantially less (Figure 1c). Only sample source (tissue or serum) and sample pathology (normal, GERD, BE, BE with LGD, or EAC) had statistically significant contributions, indicating that tissue and serum miRNA pools represent distinct expression profiles. Normal and pathological tissue samples could be easily distinguished, but that was not the case for serum samples likely because of significant measurement noise and error arising from known technical challenges of profiling miRNAs in blood (30). Thus, all further analyses only focused on HTS data from tissue miRNA profiles.

### Tissue miRNA profiles differ between normal and pathological state

We next reran principal component analysis and RDA analysis only on the 25 tissue samples. Ordination algorithms clearly separated normal samples from the rest (Figure 1d, e). Different pathological states were partially separated from each other with a significant overlap observed in several cases. EAC samples had the highest variability in miRNA profiles. Analysis of variance of RDA output indicated that sample pathology explained the highest percent of variance (Figure 1f).

### MiRNA expression does not show a gradual deviation from healthy state

**Figure 2.**
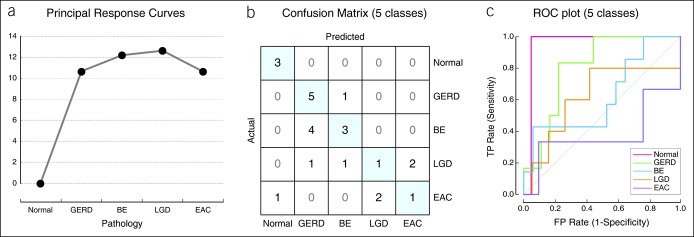
Modeling tissue miRNA expression with 5 classes. (**a**) Principal response curve (PRC) analysis of the filtered tissue miRNA data set. miRNA expression profiles in normal samples were set as baseline and were compared with the miRNA profiles of all other samples. Larger values on the y axis represent greater shift of the miRNA expression from those of normal samples. (**b**, **c**) Logistic regression (LR)-based discriminatory analysis of tissue miRNA profiles (regularization threshold C = 0.2). Each of the 5 sample types was defined as a separate class. Confusion matrix reveals the concordance of the predicted vs actual class labels of all profiled tissue samples (**b**). Receiver operating characteristic (ROC) plot (**c**) illustrates the diagnostic ability of the LR classifier as its discrimination threshold is varied. miRNA, microRNA.

We used principal response curve (PRC) analysis (31) to examine whether changes in miRNA expression profiles correlated with disease stage from normal, GERD, BE, BE with LGD, and EAC. PRC constitutes a partial RDA that isolates the sample pathology variable using normal samples as the baseline. MiRNA expression deviated from that of the healthy tissues in GERD, and that deviation did not increase in BE or BE with LGD and EAC (Figure 2a). Therefore, PRC analysis supported the hypothesis that miRNA profiles of individual pathologies are distinct classes and are not progressive stages of the same disease.

### Discriminant analysis of miRNA profiles does not support a 5 pathological state model

We first performed logistic regression-based discriminant analysis of the tissue miRNA expression data set using 5 classes (normal, GERD, BE, LGD, and EAC). Lasso regularization (LR) was used to limit the number of miRNA variables defining each class and avoid overfitting the discriminating model. The five-class model performed poorly with an accuracy of class prediction of 52% and average area under the curve (AUC) of 0.71 (Figure 2b, c). The model was able to discriminate normal samples, but could not distinguish between GERD and BE or between LGD and EAC. The overlap in miRNA expression between GERD and BE and between LGD and EAC corroborated the analyses in Figure 1d, e. MiRNA profiles of EAC samples exhibited the largest variability and thus the worst receiver operating characteristic curve (Figure 2c).

### MiRNA profiles define 3 pathology classes

**Figure 3.**
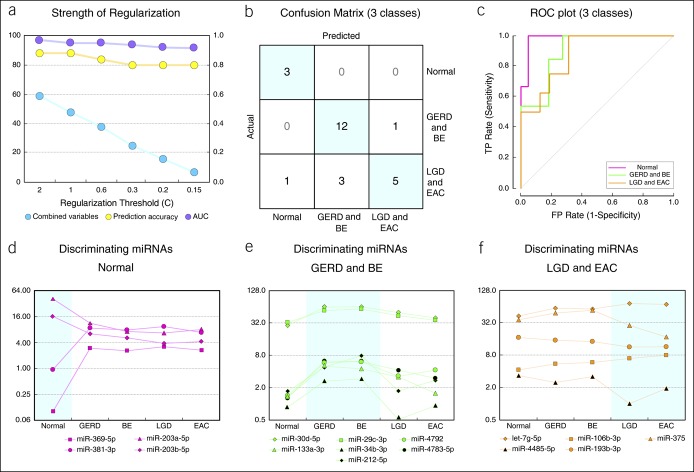
Discriminating tissue miRNA expression with 3 distinct classes. (**a**) Dependency of logistic regression (LR)-based discriminatory analysis on the strength of regularization threshold (**c**). AUC—area under the curve in ROC analysis represents the discrimination ability of each model (higher value equals better discrimination). The combined number of discriminating miRNA variables among 3 classes is plotted on the left-hand y axis; AUC and prediction accuracy values are plotted on the right-hand y axis. C = 0.2 was chosen for further model assessment. (**b**, **c**) LR discriminatory analysis of tissue miRNA profiles with 3 defined classes (Figure 2). (**d** through **f**) miRNA expression levels (arbitrary units) of discriminatory miRNAs selected by the LR algorithm to define each class. Note that y axis values are plotted on a log_2_ scale. miRNA, microRNA; ROC, receiver operating characteristic.

Because of the overlap between classes, a new logistic regression model was run with 3 classes: normal, pooled GERD and BE, and pooled LGD and EAC samples. We tested different levels of regularization strength ranging from C = 0.15 to 2 (Figure 3a) and observed only a minor improvement in prediction accuracy and AUC performance with higher numbers of discriminating miRNAs (Figure 3a). The three-class model performed better than the five-class model with a prediction accuracy of 80% and average AUC of 0.92 (Figure 3b, c). Most samples misclassified by this model were from the LGD/EAC class, consistent with the partial overlap of miRNA expression profiles in this class with the other 2 classes (Figure 1d). We chose a regularization strength cutoff of C = 0.2 as it produced the optimal number of discriminating miRNAs among the 3 classes with 4 miRNAs defining normal samples (Figure 3d), 7 miRNAs defining GERD and BE (Figure 3e), and 5 miRNAs defining LGD and EAC (Figure 3f).

### miRNAs discriminate among the 3 tissue pathology classes

**Figure 4.**
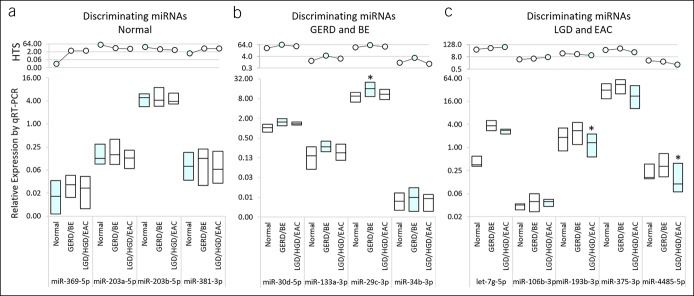
Comparison of relative miRNA expression measurements by HTS and qRT-PCR. Relative expression of miRNAs predictive of (**a**) normal, (**b**) GERD and BE, and (**c**) LGD and EAC samples is shown as determined by HTS (square-root–transformed and Loess-normalized miRNA read counts, upper row panels) and qRT-PCR from FFPE tissue samples (2^−ΔCT^ values, lower row panels). Y axes are plotted on a log_2_ scale. Box plots of 2^−ΔCT^ values obtained by qRT-PCR display median value (midline) and first and third quartile values. Light blue shading indicates the predicting class for each miRNA. *P < 0.05. BE, Barrett's esophagus. EAC, esophageal adenocarcinoma; FFPE, formalin-fixed, paraffin-embedded; GERD, gastroesophageal reflux disease; LGD, low-grade dysplasia; miRNA, microRNA; qRT-PCR, quantitative reverse transcription polymerase chain reaction.

To validate the HTS data, we used qRT-PCR to compare the expression of 16 potential predictor miRNAs (Figure 3d–f) in at least 15 FFPE samples in each of the 3 classes (n = 46 total). MiR-212-5p, miR-4792, and miR-4783-5p were undetectable by qRT-PCR and excluded from further analysis. Relative expression (2^−ΔCT^) of the remaining 13 miRNAs (Figure 4, lower panels) and the corresponding miRNA read abundances measured by HTS (Figure 4, upper panels) are shown. Two of the 4 miRNA predictors of normal samples, miR-369-5p and miR-203b-5p, showed similar trends in median expression by qRT-PCR as observed by HTS (Figure 4a), while the trends for miR-203a-5p and miR-381-3p differed from those observed by HTS. Although all 4 miRNA predictors of GERD and BE (miR-133a-3p, miR-29c-3p, miR-30d-5p, and miR-34b-3p) showed an increase in median expression relative to normals by qRT-PCR as observed by HTS (Figure 4b), only miR-29c-5p reached statistical significance (*P* = 0.043). MiRNA predictors of LGD and EAC showed similar trends by qRT-PCR with minor deviations observed for GERD/BE samples for miR-193b-5p and miR-4485-5p (Figure 4c). Both miR-193b-3p and miR-4485-5p showed significantly different expression levels vs normal and GERD/BE classes (*P* = 0.005 and 0.014, respectively).

### Target analysis of miRNA predictors of esophageal pathology

We used IPA to identify 36 validated mRNA targets of the 16 miRNAs identified in this study (Figure 3d–f) as shown in Table 3. Three of these mRNA targets, COL1A2, DAD1, and CCND1, were previously shown to be associated with BE or EAC (32–34). The remaining mRNA targets have not been previously associated with BE or EAC. Overall, the mRNAs targets in Table 3 showed enrichment for relevant cellular functions including fibrosis, cell movement, colony formation, and cell invasion (all *P* < 0.0001).

**Table 3.**
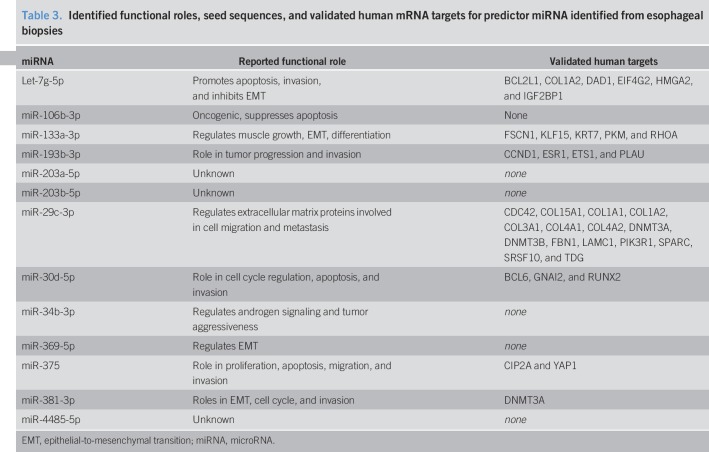
Identified functional roles, seed sequences, and validated human mRNA targets for predictor miRNA identified from esophageal biopsies

## DISCUSSION

The histological presence of dysplasia in esophageal biopsies is frequently overlooked because of sampling bias during endoscopy and poor interobserver agreement (35,36), thus necessitating improved methods for staging disease and risk. Models that incorporate clinical and histologic data with biomarkers show improved predictive capacity (37). Our study used HTS profiling of miRNA from serum and esophageal tissue biopsies to identify miRNAs predictive for normal, GERD/BE, and/or LGD/HGD/EAC pathology classes. Sixteen miRNAs were identified which successfully classified biopsy samples into 3 classes (normal, GERD and BE, and BE with LGD and EAC) with a prediction accuracy of 80%. qRT-PCR confirmed the relative expression of 11 miRNAs, providing an miRNA signature with potential clinical utility as staging biomarkers. These miRNAs could be used to improve diagnosis when used as an adjunct to current histopathology. Moreover, the cost associated with this added screening would be kept to a minimum since tissue miRNA levels may be assessed from existing FFPE samples collected during normal histologic screening. Finally, the identification of downstream mRNA targets of these miRNAs and their function will provide critical insights into the pathophysiology of esophageal cancer.

The importance of incorporating relevant clinical data into mathematical models is well documented (38). This study used RDA (Figure 1c) to account for the effect of BMI, age, and sample pathology as confounding variables. Most variance between the samples used for HTS was attributed to sample pathology, while age and BMI had a smaller influence (Figure 1f). Although previous studies have shown a correlation between high BMI and EAC (39), the trend was not observed in our data set. The effect of sex was not incorporated into the RDA model since all the NGS samples were acquired from male patients (Table 1). This sampling is consistent with previous reports that esophagitis, BE, and EAC are more common in male patients (40). Although this sample distribution rules out sex as a confounding variable in our analysis, it is possible that some of the changes in miRNA expression may be male-specific. Additional testing would be required to determine whether similar changes in miRNA expression are observed in esophageal biopsies from female patients. Finally, consistent with previous reports that cigarette smoking is a risk factor for EAC (41), we observed that a majority of normal biopsies were collected from nonsmokers, while a majority of samples in the LGD/HGD/EAC group were from smokers. Altogether, our analysis indicates that pathology explained a majority of the variance in our HTS data set. An increase in sample size would be required to determine the effect of other confounding variables.

Previous reports have reported a 3- to 7-fold increase in the rate of disease progression in patients with long-segment BE compared with short-segment BE (42,43). All but one of the samples used in this study were obtained from patients with long-segment BE, thus excluding segment length as a confounding variable. Analysis of miRNA levels from longitudinally collected samples including a mix of short- and long-segment BE would be required to identify miRNA which predict patients at risk of disease progression.

The extent to which circulating biomarkers reflect changes at the affected tissue level is unclear. Sierzega et al. (44) found that only 7 of 20 circulating miRNAs upregulated in gastric cancer were overexpressed in primary tumors, suggesting elevated miRNA levels may not originate from primary tumors. This discordance between circulating and tissue miRNA levels has also been observed in response to acute resistance exercise (45). Our study failed to identify a serum miRNA signature of disease state, thus suggesting that miRNA may have limited ability to distinguish between disease states and limiting the utility of circulating miRNA in early disease detection. Furthermore, our analysis supports a disconnect between serum and tissue mRNA profiles since miRNA levels measured from esophageal tissue biopsies correctly characterized samples into normal, BE/GERD, or LGD/EAC groups with 80% accuracy. The three-class model used in this study yielded an accuracy of 80% (AUC = 0.92), in line with previous BE studies. Duits et al. (46) reported an AUC of 0.73 for their model using histology and 3 mRNA biomarkers to predict progression to EAC. Eluri et al. (47) reported an accuracy of 89.9% (AUC = 0.95) for their model using mutation load to predict disease progression.

The predictive power of the model relies only on the association of altered miRNA levels with a specific disease stage(s), thus inferences related to the role these miRNAs play in disease progression cannot be made. Furthermore, it is unclear from the predictive model whether the observed changes in miRNA expression are drivers or readouts of disease pathology. Nonetheless, the miRNA identified by this model and the validated mRNA targets of these miRNAs provide information which is critical to the understanding of miRNA in esophageal cancer physiology. Future studies using samples taken over time from the same individual could be used identify miRNA biomarkers of disease progression.

Our analysis identified a 13 miRNA signature of esophageal disease from normal tissue, GERD and BE, and LGD and EAC. Additional insights into the functional role played by these miRNAs in esophageal cancer may be made by correlating our HTS data with studies focused on known mRNA targets (Table 1). For example, miR-29c-3p is upregulated only in BE but not EAC (48) and is shown to target 10 extracellular matrix proteins involved in cell migration and metastasis in HeLa and HepG2 cells, suggesting it can play a role in the cell migration and metastasis observed in EAC (49).

Eleven of the 13 miRNAs tested by qRT-PCR showed matching profiles of expression between classes when compared with the HTS data further validating the HTS data. Our results demonstrated a significant upregulation of miR-29c-3p in BE and downregulation of miR-193b-3p in EAC consistent with previously published studies (50,51). In addition, we observed that miR-133a-3p and miR-106b are upregulated in both BE and EAC, and that miR-375 is upregulated in BE and downregulated in EAC as shown previously (48,52–54).

Let-7g-5p was shown to be upregulated in GERD/BE and LGD/EAC samples relative to normal controls (Figure 4). Although Let-7g-5p is upregulated in advanced renal cell carcinoma (55) and associated with recurrence in lung adenocarcinoma (56), it has not been previously associated with esophageal disease, thus identifying it as a potential novel biomarker of early esophageal disease. Let-7g-5p directly targets BCL2L1 (BCL-X) (57), and loss of BCL2L1 has been associated with progression to EAC and reduced survival (58,59). In addition, Let-7g-5p has also been shown to target high mobility group AT-hook 2 (HMGA2), IGF2BP1 (IMP-1), and COL1A2 previously shown to be associated with EAC (60–63).

MiR-193b-3p was significantly downregulated in LGD/EAC samples relative to normal controls by HTS and qRT-PCR (*P* = 0.0141), consistent with previous reports (50,51). Loss of miR-193b-3p leads to elevated uPA levels and increases breast cancer progression and invasion (64), but a role for uPA in BE or EAC has not been reported. MiR-193b-3p targets ERα (ESR1) (65) which is hypermethylated in GERD, BE, and EAC (66), suggesting that miR-193b-3p and epigenetic silencing may act in parallel to silence ESR1. MiR-193b-3p also targets CCND1 and ETS1 to induce cell cycle arrest and inhibit migration and invasion (67). Polymorphisms in CCND1 are associated with increased risk of GERD (68).

MiR-30d-5p was upregulated in all pathologies relative to normal controls by HTS and qRT-PCR. Although dysregulation of miR-30d-5p occurs in colon and non–small cell lung cancer, its association with BE or EAC has not been reported (69,70). MiR-30d-5p directly targets BCL6 (71), and BCL6 downregulation leads to increased cyclin D2 expression and invasion (72,73), suggesting that miR-30d-5p may regulate cell cycle progression and metastasis in EAC. MiR-30d-5p also targets the proto-oncogene Galphai2 (GNAI2) (74), but altered GNAI2 levels in EAC have not been reported. Finally, miR-30d-5p also targets RUNX2 (70) which has been linked to BE to EAC progression (75).

MiR-375 was downregulated in LGD/HGD/EAC samples relative to GERD/BE samples by HTS and qRT-PCR. These results are consistent with previous reports linking miR-375 to BE to EAC progression (48,52,76). Downregulation of miR-375-3p increases yes-associated protein (YAP) (77), a marker upregulated in gastric cancer associated with poor prognosis (78). YAP1 is overexpressed in EAC cell lines relative to BE cell lines (79), thus suggesting that loss of miR-375 in EAC may promote proliferation and invasion of cancer cells. MiR-375 regulates c-MYC by targeting Cancerous Inhibitor of PP2A (CIP2A) (80), both of which are overexpressed in BE and EAC (81). Finally, miR-375 targets YWHAZ (14-3-3ζ) which regulates proliferation, apoptosis, migration, and invasion in gastric cancer cell lines (82).

MiR-4485-5p was significantly downregulated in the LGD/HGD/EAC class relative to normal and GERD/BE classes, thus potentially serving as a novel marker of disease severity. Although dysregulation of miR-4485 in esophageal disease has not been reported, it is a known tumor suppressor that binds to mitochondrial 16S rRNA and regulates mitochondrial complex 1 activity leading to altered ATP production, caspase activation, and apoptosis (83). Interestingly, miR-4485 is a target of NF-κB signaling which is downregulated in TNFα-stimulated HeLa cells (84). The significant downregulation of miR-4485 observed in this study may thus be a result of the typical elevation of NF-κB signaling observed in GERD, BE, and EAC (21).

Taken together, these miRNAs provide a signature of normal, GERD ,and BE and dysplastic (i.e., LGD, HGD, and EAC) pathological states. Furthermore, the dysregulation of these miRNA may provide critical insights into the specific cellular physiology changes that occur in Barrett's esophagus and EAC.

## CONFLICTS OF INTEREST

**Guarantor of the article**: Madhavi P. Kadakia, PhD.

**Specific author contributions:** M.P.K. and S.A.: study concept and design. S.M., S.A., and M.S.: sample procurement and histologic analysis. M.P.K., J.Z., N.R., and M.P.C.: acquisition of data. M.P.K., M.P.C., O.P., and M.B.: analysis and interpretation of data. M.P.C., O.P., M.B., S.A., and M.P.K.: critical revision of the manuscript. M.B. and O.P.: statistical analysis. M.P.K. and S.A.: study supervision.

**Financial support:** Funding was provided through a pilot grant from Wright State University and Dayton VA Medical Center to M.P.K. and S.A.

**Potential competing interests:** None to report.

**Study Highlights**

**WHAT IS KNOWN**

✓ Altered expression of serum and tissue miRNAs is observed in Barrett's esophagus and esophageal adenocarcinoma.
✓ Biomarkers of Barrett's esophagus and esophageal adenocarcinoma are needed to improve disease staging.

**WHAT IS NEW HERE**

✓ Approximately 16 miRNAs were identified which successfully classified biopsy samples from normal, Barrett's esophagus/gastroesophageal reflux disease, and esophageal adenocarcinoma subjects with an accuracy of 80%.
✓ Using quantitative reverse transcription polymerase chain reaction, the relative expression of 11 miRNAs was confirmed providing an miRNA signature with potential clinical utility.

## Supplementary Material

SUPPLEMENTARY MATERIAL
