# Supplementary material for: Differential MicroRNA Signatures in the Pathogenesis of Barrett's Esophagus
Source: Clin Transl Gastroenterol. 2020 Jan 13;11(1):e00125. doi: 10.14309/ctg.0000000000000125 (PMC7056055; doi:10.14309/ctg.0000000000000125)
Supplement: SUPPLEMENTARY MATERIAL [file ct9-11-e00125-s001.docx]

**Supplemental Table 1: Detailed clinical features of the subjects used for HTS sequencing**

| Sample | Age | Gender | BMI | Smoking History | Years with BE |
| --- | --- | --- | --- | --- | --- |
| Normal A | 80 | M | 23 | N | N/A |
| Normal B | 39 | M | 20 | Y | N/A |
| Normal C | 69 | M | 31 | Y | N/A |
| Normal D | 55 | M | 26 | N | N/A |
| GERD A | 76 | M | 29 | N | N/A |
| GERD B | 71 | M | 30 | Y | N/A |
| GERD C | 88 | M | 26 | N | N/A |
| GERD D | 67 | M | 31 | N | N/A |
| GERD E | 52 | M | 31 | Y | N/A |
| GERD F | 69 | M | 31 | N | N/A |
| GERD G | 68 | M | 30 | N | N/A |
| GERD H | 60 | M | 29 | N | N/A |
| BE A | 58 | M | 30 | N | 0 |
| BE B | 73 | M | 26 | N | 3.6 |
| BE C | 45 | M | 26 | Y | 0 |
| BE D | 81 | M | 24 | N | 1.2 |
| BE E | 66 | M | 29 | N | 3.1 |
| BE F | 79 | M | 26 | Y | 0.2 |
| BE G | 63 | M | 34 | N | 5.5 |
| LGD A | 76 | M | 32 | Y | 11.2 |
| LGD B | 70 | M | 34 | Y | 0.1 |
| LGD C | 74 | M | 24 | Y | 11.5 |
| LGD D | 59 | M | 32 | N | 0 |
| LGD E | 51 | M | 29 | Y | 8.7 |
| EAC F | 65 | M | 27 | N | Unknown |
| EAC G | 46 | M | 20 | Y | Unknown |
| EAC H | 58 | M | 17 | Y | Unknown |
| EAC I | 70 | M | 40 | N | 8.7 |
| EAC J | 57 | M | 41 | N | 0 |

N/A: Normal or GERD samples for which “years with BE” is not applicable

Unknown: No diagnosis date for BE available, so time with BE is unknown.
