# Supplementary material for: Differential MicroRNA Signatures in the Pathogenesis of Barrett's Esophagus
Source: Clin Transl Gastroenterol. 2020 Jan 13;11(1):e00125. doi: 10.14309/ctg.0000000000000125 (PMC7056055; doi:10.14309/ctg.0000000000000125)
Supplement: SUPPLEMENTARY MATERIAL [file ct9-11-e00125-s002.docx]

**Supplemental Table 2: Detailed clinical features of the FFPE samples from subjects used for qRT-PCR validation**.

| \| Sample \| Age \| Gender \| BMI \| Smoking History \| Years with BE \| \| --- \| --- \| --- \| --- \| --- \| --- \| \| Normal 1 \| 62 \| M \| 33 \| No \| N/A \| \| Normal 2 \| 68 \| M \| 36 \| No \| N/A \| \| Normal 3 \| 55 \| M \| 29 \| Yes \| N/A \| \| Normal 4 \| 72 \| M \| 18 \| No \| N/A \| \| Normal 5 \| 65 \| M \| 25 \| No \| N/A \| \| Normal 6 \| 39 \| M \| 29 \| No \| N/A \| \| Normal 7 \| 55 \| M \| 30 \| No \| N/A \| \| Normal 8 \| 53 \| M \| 29 \| Yes \| N/A \| \| Normal 9 \| 63 \| M \| 18 \| No \| N/A \| \| Normal 10 \| 53 \| M \| 29 \| Yes \| N/A \| \| Normal 11 \| 68 \| M \| 20 \| Yes \| N/A \| \| Normal 12 \| 65 \| M \| 26 \| Yes \| N/A \| \| Normal 13 \| 65 \| F \| 30 \| No \| N/A \| \| Normal 14 \| 62 \| M \| 18 \| No \| N/A \| \| Normal 15 \| 52 \| M \| 28 \| No \| N/A \| \| GERD 1 \| 56 \| M \| 38 \| Yes \| N/A \| \| GERD 2 \| 68 \| M \| 33 \| No \| N/A \| \| GERD 3 \| 66 \| M \| 31 \| No \| N/A \| \| GERD 4 \| 57 \| M \| 30 \| No \| N/A \| \| GERD 5 \| 61 \| M \| 27 \| Yes \| N/A \| \| GERD 6 \| 71 \| M \| 35 \| No \| N/A \| \| GERD 7 \| 70 \| M \| 20 \| Yes \| N/A \| \| GERD 8 \| 56 \| M \| 26 \| No \| N/A \| \| BE 1 \| 64 \| M \| 28 \| Yes \| Unknown \| \| BE 2 \| 66 \| M \| 28 \| Yes \| Unknown \| \| BE 3 \| 57 \| M \| 34 \| No \| 0.8 \| \| BE4 \| 68 \| M \| 44 \| No \| Unknown \| \| BE5 \| 66 \| M \| 28 \| Yes \| Unknown \| \| BE6 \| 68 \| M \| 43 \| No \| Unknown \| \| BE7 \| 72 \| M \| 30 \| No \| Unknown \| \| BE8 \| 64 \| M \| 43 \| Yes \| 4.1 \| \| LGD 1 \| 61 \| M \| 32 \| No \| 3.37 \| \| LGD 2 \| 70 \| M \| 30 \| Yes \| Unknown \| \| LGD 3 \| 73 \| M \| 23 \| Yes \| 10.5 \| \| LGD 4 \| 83 \| M \| 23 \| No \| 9.6 \| \| LGD 5 \| 74 \| M \| 24 \| Yes \| 11.5 \| \| LGD 6 \| 58 \| M \| 31 \| No \| 17.66 \| \| LGD 7 \| 67 \| M \| 29 \| No \| 3.85 \| \| HGD 1 \| 81 \| M \| 22 \| No \| Unknown \| \| EAC 1 \| 70 \| M \| 30 \| Yes \| 0.9 \| \| EAC 2 \| 71 \| M \| 18 \| Yes \| Unknown \| \| EAC 3 \| 61 \| M \| 31 \| yes \| 9.08 \| \| EAC 4 \| 76 \| M \| 22 \| Yes \| 9.82 \| \| EAC 5 \| 81 \| M \| 18 \| Yes \| 6.05 \| \| EAC 6 \| 60 \| M \| 19 \| Yes \| Unknown \| \| EAC 7 \| 94 \| M \| 22 \| No \| Unknown \| |  |  |  |  |  |
| --- | --- | --- | --- | --- | --- | --- | --- | --- | --- | --- | --- | --- | --- | --- | --- | --- | --- | --- | --- | --- | --- | --- | --- | --- | --- | --- | --- | --- | --- | --- | --- | --- | --- | --- | --- | --- | --- | --- | --- | --- | --- | --- | --- | --- | --- | --- | --- | --- | --- | --- | --- | --- | --- | --- | --- | --- | --- | --- | --- | --- | --- | --- | --- | --- | --- | --- | --- | --- | --- | --- | --- | --- | --- | --- | --- | --- | --- | --- | --- | --- | --- | --- | --- | --- | --- | --- | --- | --- | --- | --- | --- | --- | --- | --- | --- | --- | --- | --- | --- | --- | --- | --- | --- | --- | --- | --- | --- | --- | --- | --- | --- | --- | --- | --- | --- | --- | --- | --- | --- | --- | --- | --- | --- | --- | --- | --- | --- | --- | --- | --- | --- | --- | --- | --- | --- | --- | --- | --- | --- | --- | --- | --- | --- | --- | --- | --- | --- | --- | --- | --- | --- | --- | --- | --- | --- | --- | --- | --- | --- | --- | --- | --- | --- | --- | --- | --- | --- | --- | --- | --- | --- | --- | --- | --- | --- | --- | --- | --- | --- | --- | --- | --- | --- | --- | --- | --- | --- | --- | --- | --- | --- | --- | --- | --- | --- | --- | --- | --- | --- | --- | --- | --- | --- | --- | --- | --- | --- | --- | --- | --- | --- | --- | --- | --- | --- | --- | --- | --- | --- | --- | --- | --- | --- | --- | --- | --- | --- | --- | --- | --- | --- | --- | --- | --- | --- | --- | --- | --- | --- | --- | --- | --- | --- | --- | --- | --- | --- | --- | --- | --- | --- | --- | --- | --- | --- | --- | --- | --- | --- | --- | --- | --- | --- | --- | --- | --- | --- | --- | --- | --- | --- | --- | --- | --- | --- | --- | --- | --- | --- | --- | --- | --- | --- | --- | --- | --- | --- |

N/A: Normal or GERD samples for which “years with BE” is not applicable

Unknown: No diagnosis date for BE available, so time with BE is unknown.
